# Breath-by-Breath Measurement of Respiratory Frequency and Tidal Volume with a Multiple-Camera Motion Capture System During Cycling Incremental Exercise

**DOI:** 10.3390/s25082578

**Published:** 2025-04-19

**Authors:** Carlo Massaroni, Andrea Nicolò, Ana Luiza de Castro Lopes, Chiara Romano, Mariangela Pinnelli, Karine Sarro, Emiliano Schena, Pietro Cerveri, Massimo Sacchetti, Sergio Silvestri, Amanda Piaia Silvatti

**Affiliations:** 1 Unit of Measurements and Biomedical Instrumentation, Departmental Faculty of Engineering, Università Campus Bio-Medico di Roma, 00128 Rome, Italy; c.romano@unicampus.it (C.R.); mariangela.pinnelli@unicampus.it (M.P.); e.schena@unicampus.it (E.S.); s.silvestri@unicampus.it (S.S.); 2 Fondazione Policlinico Campus Bio-Medico di Roma, 00128 Rome, Italy; 3 Department of Movement, Human and Health Sciences, University of Rome “Foro Italico”, 00135 Rome, Italy; andrea.nicolo@uniroma4.it (A.N.); massimo.sacchetti@uniroma4.it (M.S.); 4 Faculdade de Educação Física, Universidade Estadual de Campinas, Campinas 13083-970, Brazil; analuiza.castrolopes@gmail.com (A.L.d.C.L.); ksarro@unicamp.br (K.S.); 5 Dipartimento di Elettronica Informazione e Bioingegneria, Politecnico di Milano, 20133 Milano, Italy; pietro.cerveri@polimi.it; 6 Department of Industrial and Information Engineering, University of Pavia, Via Adolfo Ferrata 5, 27100 Pavia, Italy; 7 Departamento de Educação Física, Universidade Federal de Viçosa, Viçosa 36570-900, Brazil

**Keywords:** measurements, breathing biomechanics, motion capture systems, incremental exercise, breathing monitoring, validity

## Abstract

This study evaluates the performance of a 32-marker motion capture (MoCap) system in estimating respiratory frequency ($f_{R}$) and tidal volume ($V_{T}$) during cycling exercise. Fourteen well-trained cyclists performed an incremental step test on a cycle ergometer, while simultaneously recording a raw flow signal with a reference metabolic cart (COSMED) and respiratory-induced torso movements with twelve optoelectronic cameras registering the position of 32 markers affixed to the torso. $f_{R}$ and $V_{T}$ were calculated from both systems on a breath-by-breath basis. The MoCap system showed a strong correlation with the COSMED system when measuring $f_{R}$ and $V_{T}$ ($r^{2}$ = 0.99, $r^{2}$ = 0.87, respectively) during exercise. For $f_{R}$, the mean absolute error (MAE) and mean absolute percentage error (MAPE) were 0.79 breaths/min and 2.1%, respectively. For $V_{T}$, MoCap consistently underestimated values compared to COSMED, showing a bias (MOD ± LOA) of −0.11 ± 0.42 L and MAPE values of 8%. These findings highlight the system’s capabilities for real-time respiratory monitoring in athletic environments.

## 1. Introduction

Optical motion capture systems (MoCap) are used in numerous fields, such as sports biomechanics [1], gait analysis [2], and rehabilitation of motor dysfunctions [3]. These systems use a set of fixed cameras that need to be calibrated and capture the scene while the subject moves. The sequence of frames is then processed to extract information about the subject’s movement. Two categories are distinguished: markerless optical systems and marker-based optical systems. Markerless optical systems do not require the placement of reflective markers on the subject. These systems are less mature compared to marker-based systems and are based on the analysis of 2D images captured by cameras. Marker-based systems are more accurate and can be further divided into systems that use active or passive markers [4].

Both marker-based and markerless systems have been explored for the measurement of respiratory variables and ventilatory volumes. One of the markerless optical systems used to assess tidal volume is structured light plethysmography [5]. It is a non-invasive technique that uses structured light to indirectly measure respiratory activity without any contact with the subject under examination. Although structured light plethysmography is validated for measuring respiratory activity and ventilatory volumes without the use of markers on the chest wall, it can only be used for quiet and natural breathing, with the subject having to be seated or in a supine position and must remain still throughout the test and only captures the front part of the chest wall, assuming the absence of movement of the posterior wall. As for other markerless systems based on optical measurement [6], even minimal movement of the subject from the reference plane significantly compromises data collection.

By placing retro-reflective markers on the surface of the chest wall in predetermined anatomical landmarks, it is possible to indirectly measure the pulmonary volumes of a subject [7]. The system consists of user-surrounding cameras that emit infrared rays, which are reflected by markers placed on a subject. Variations in trajectories over time of individual points recorded by the camera-connected workstation allow for the indirect measurement of compartmental tidal volumes (hereafter $V_{T}$) by registering the movements of the chest wall [8]. The chest wall can be divided into three independent compartments: pulmonary rib cage (RCp), the abdominal rib cage (RCa), and the abdomen (AB) [9,10]. Marker-based motion capture systems with multiple cameras enable the measurement of the volume of each compartment. Another advantage is its non-invasiveness, as it does not create pneumatic resistance during measurement (as in the case of pneumotachographs). Additionally, these systems provide the opportunity to perform the measurement even when the user moves.

From a technical point of view, six or more cameras are needed to estimate volumes in numerous postures. In a generic clinical application context, four to eight infrared cameras and IR-reflective hemispheric or spherical markers, with a diameter ranging from 6 mm to 10 mm, have typically been used. In general, a smaller marker size facilitates more precise reconstruction, especially in protocols with multiple markers. This is because the larger the marker size, the greater the distance between the marker and the point of marker application on the subject’s skin, resulting in a shifted trajectory relative to the movement of the skin.

The use of a specific set of markers is essential for $V_{T}$ estimation. The most used are protocols with 89 markers (i.e., a full marker set) [8], reduced protocols based on 32 markers [11], protocols with 52 markers for assessing subjects in the supine position as in intensive care [12], and protocols with 24 markers for analyzing respiratory volumes in newborns in supine posture, and those which involve marker placement on only one side of the torso [13]. The protocol referred to as the standard provided a more accurate reconstruction of the chest wall surface compared to other protocols. It was initially developed with 86 markers and demonstrated good accuracy both at rest and during movement [7,10,14]. Later, this protocol was refined to a composition of 89 markers [15], with a maximum measurement error on the tidal volume estimation below 4% on average values calculated over 180 s of maximal exercise [16].

Despite the good accuracy of the standard protocol, the use of alternative protocols is more feasible in some applied settings. For instance, the protocol with 32 markers has been used in both clinical and sports settings [17,18], as it offers several advantages. It is an easier and faster protocol that enhances measurement reproducibility and facilitates the rapid processing of data under various environmental conditions. Furthermore, the reduced number of markers on the trunk enables its use in various conditions, including the cyclist’s natural position. Conversely, the studies mentioned above that used the 89-marker protocol during exercise were conducted with participants seated upright on the bicycle, with their hands off the handlebars. An underestimation of $V_{T}$ has been reported when assessing the performance of the 32-marker protocol compared to the 89-marker protocol during quiet breathing. Massaroni and colleagues [19] found that the average percentage discrepancy between the two protocols was 1.71 ± 1.23% in the range of 0.35 L to 2.78 L. However, we are not aware of studies validating the 32-marker protocol during cycling exercise.

Therefore, the purpose of this study was to investigate the breath-by-breath performance of the MoCap system using the 32-marker protocol during cycling exercise. Well-trained competitive cyclists performed an incremental exercise test while measuring $f_{R}$ and $V_{T}$ with a MoCap system and a reference system simultaneously. To improve the ecological validity of the study findings, cyclists were tested in the posture they commonly use when riding a bike.

## 2. Materials and Methods

### 2.1. Marker-Based Motion Capture System (MoCap)

**Figure 1:**
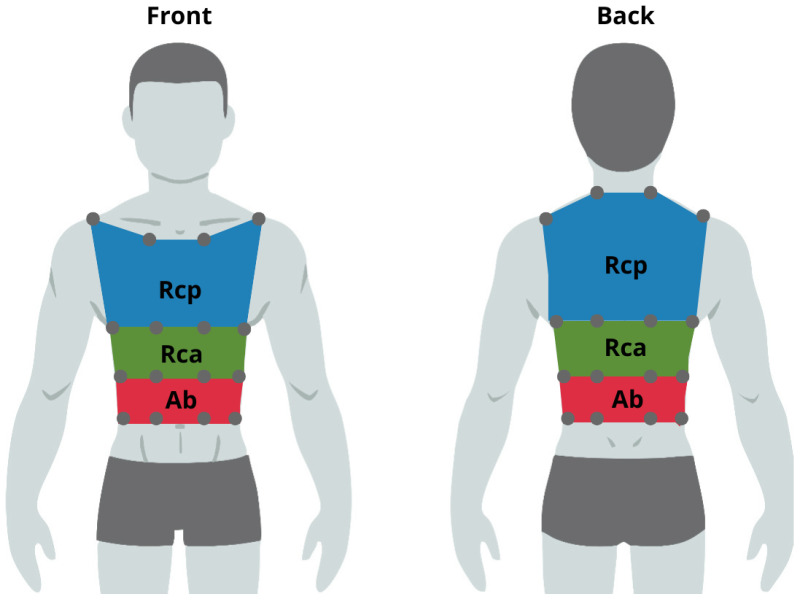
Markers arrangement: The blue, green, and red areas represent, respectively, the RCp, RCa, and AB compartments.

To collect tridimensional kinematic data, we used twelve OptiTrack Prime 17 W cameras (NaturalPoint, Corvallis, OR, USA) with an acquisition frequency of 360 Hz, autofocus, a wide-angle (FOV) of 70°, a resolution of 1.7 megapixels, and an accuracy better than 1 mm [20]. The markers used have a spherical shape because this shape allows for better reflection of the infrared rays emitted by the cameras. The markers chosen for this application are passive, which has the advantage of being cable-free and reducing clutter in the test environment. They provide greater freedom of movement for the tested subjects and do not require any additional information exchange with the system’s core before being used. A hypoallergenic adhesive layer is placed under the base of the markers, and they are placed directly in contact with the subject’s skin. The protocol, composed of 32 markers for respiratory biomechanics, was chosen to divide the thoracic cage into three compartments (RCp, RCa, and AB). Each compartment is further divided into three sub-volumes according to the studies by Ferrigno et al. [11] (Figure 1). This protocol divides the chest wall into four horizontal lines (at the level of second rib, xiphoid process, tenth rib, and abdominal transversal line) and four vertical lines equally spaced from the midline, between the anterior axillary line and the mid-axillary line, positioned on the anterior and posterior regions of the trunk. The RCp reflects the action of the neck and parasternal muscles, as well as pleural pressure; the RCa reflects the action of the diaphragm, along with pleural and intra-abdominal pressures; and the AB reflects the action of the diaphragm and abdominal muscles. Markers’ trajectories were reconstructed using the software Motive^®^ Optitrack Software v.2.1 (NaturalPoint, Corvallis, OR, USA) and exported as .csv format.

In MATLAB (version R2024a) volume signals from the MoCap system were calculated using the previously validated Convex Hull method with boundary conditions [21]. The volume is enclosed by 32 markers, ideally representing a parallelepipedal structure composed of three respiratory compartments. Each compartment is further subdivided into three sub-volumes, resulting in a total of nine volume units. Each unit was enclosed by eight markers (four anterior and four posterior), and its volume is computed at each time point as the sum of the volumes of six prisms. Therefore, the total thorax volume is defined as the sum of all 54 prism volumes.

### 2.2. COSMED Reference System

A COSMED metabolic cart (Quark CPET, COSMED S.r.l., Rome, Italy) was used as the reference system to evaluate the performance of the MoCap system. The raw respiratory flow signal was recorded at 50 Hz using the COSMED flow-meter. The $V_{T}$ was calculated by integrating the flow signal. The pneumotachograph present in the equipment is based on a digital turbine with a diameter of 28 mm and a flow and ventilation range of 0.08 L/s–20 L/s and 0.08 L/min–300 L/min, respectively, with a precision of ±20 mL/s and ±200 mL/min [22]. The participants wore a face mask both during the incremental test and the quiet breathing assessment.

### 2.3. Populations

**Figure 2:**
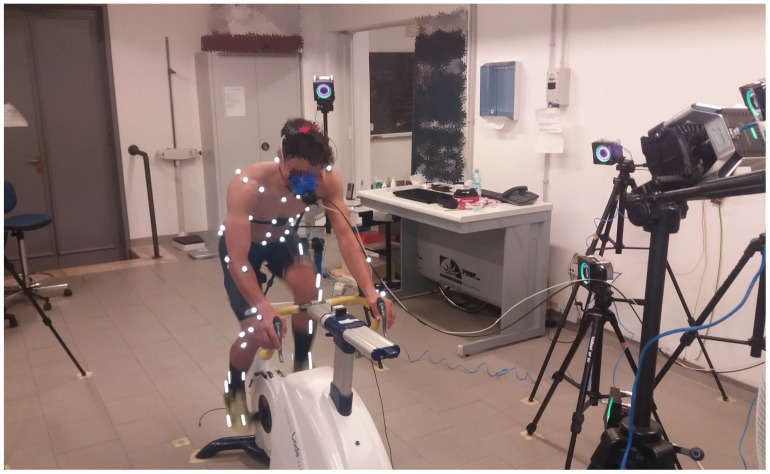
Camera setup used for testing the performance of the MoCap system.

Fourteen well-trained male cyclists (age 23 ± 4 years, body mass 63 ± 7 kg, height 1.73 ± 0.07 m, body mass index 21.1 ± 1.8 m·kg^−2^), with a minimum of 400 km of training per week, volunteered to participate in the study. They performed a step incremental test on a cycle-ergometer to assess the performance of the MoCap system during cycling exercise as detailed below (see Figure 2). The performance of the MoCap system was also assessed at rest during quiet breathing, and seven untrained male volunteers (age 27 ± 3 years, body mass 71 ± 12 kg, height 1.75 ± 0.06 m, body mass index 23.2 ± 2.5 m·kg^−2^) were recruited for this purpose.

#### Experimental Trials and Protocol

The cyclists underwent an incremental step test to exhaustion. The first step of the test consisted of 180 s at 50 W to allow further warm-up and synchronization between the instruments. Thereafter, an additional increase of 25 W every 60 s was provided. The exercise ended when the participant was no longer able to maintain a pedaling cadence above 60 revolutions per minute (rpm).

The cycle ergometer used for the incremental test is the Lode Excalibur cycle ergometer (Excalibur Sport, Lode, The Netherlands) [23]. It is a device that can be configured through its corresponding software (Excalibur Sport, Lode, The Netherlands). It has a selectable load range from 8 W to 2500 W, where the maximum load set for long-duration tests is 1500 W. In addition, the accuracy of the pedal resistance is 2 W up to 100 W and 2% from 100 W to 1500 W. The applied load is independent from pedaling cadence (within the range of 25 to 185 rpm) when the ergometer is set in hyperbolic mode. Participants were required to keep their hands in a fixed position on the handlebars and could not stand up on the pedals or detach from the saddle. Additionally, the settings of the athletes’ own bikes were accurately reproduced on the cycle ergometer.

The untrained volunteers performed 300 s of quiet breathing while resting in an upright posture. During the tests, all the participants were instructed not to speak to avoid compromising the measurements obtained from the reference and MoCap devices. The data collected on cyclists assessed the performance of the MoCap system for broad ranges of $f_{R}$ and $V_{T}$ values, while the data collected on untrained individuals complemented this evaluation by providing information on the system’s performance when breathing at rest with low values of $f_{R}$ and $V_{T}$.

### 2.4. Data Analysis

The raw data collected from the MoCap and COSMED systems were processed in the MATLAB environment. As explained below, synchronization was performed to align the COSMED volume signal and the MoCap volume signal, which was reconstructed by computing compartmental and chest wall volumes from marker trajectories.

#### 2.4.1. Synchronization Protocol

During both cycling and quiet breathing assessments, the synchronization procedure for the two systems was performed before the test, as follows. The protocol consists of a 30 s phase of quiet breathing, followed by 10 s of apnea. After this, the participant performs 10 breathing cycles, followed by another 10 s of apnea. The test begins immediately after the second apnea.

The first subsequent breath after the initial apnea is manually identified on the signals for both the COSMED and MoCap devices. In addition, a second apnea is included to ensure that the signals can be synchronized in case a clearly recognizable respiratory peak is absent after the first apnea.

#### 2.4.2. Breath Identification

From the global volume of the chest wall reconstructed from the MoCap trajectories, all minimum points on the recorded signal were used to segment each breath. A double level of control has been introduced to improve the identification of breaths. Around the signal of the total volume of the chest wall, after applying the detrend function, a square wave Q is constructed with an amplitude of | 1 |. It has a positive value during the positive phase of the signal and a negative value during the opposite phase:

(1) $$Q\left(f\right)=\left\{\begin{array}{cc} 1, & \mathrm{if}\;V_{T}\left(f\right)\geq 0\\ -1, & \mathrm{if}\;V_{T}\left(f\right)<0 \end{array}\right.$$

For each negative phase of the square wave, the corresponding part of the signal is selected, and the absolute minimum point is obtained. Similarly, for each positive phase of the square wave, the corresponding part of the signal is selected. Unlike the negative phase, a new square wave is reconstructed on the segment with a zero mean value of the signal. This is performed to check for the presence of minima that would otherwise be excluded and not counted as respiratory acts.

#### 2.4.3. Tidal Volume Calculation

The sum of the compartmental volumes represents the volume signal estimated by the MoCap system, while the integral of the flow signal represents the volume signal measured by the COSMED. On a breath-by-breath basis, for both the reference and MoCap systems, the volume signal is cut into the minimum points. Within each detected breath, the maximum point of the curve is identified to mark the end of inspiration. The difference between the volume assumed at the maxima and minima points corresponds to the $V_{T}$ of the respiratory breath.

#### 2.4.4. Respiratory Frequency Calculation

From the identified minimum points, the associated time intervals for each breath ($\Delta T_{i}$) are also calculated as the time elapsed from two consecutive minimum points. Then the breath-by-breath $f_{R}$ ($f_{R,i}$) is calculated as in the following formula:

(2) $$f_{R_{,i}}=\frac{60}{\Delta T_{i}}$$

#### 2.4.5. Compartmental Contribution

The respiratory contribution of compartments during respiration can be calculated using the chest wall protocol described before, only for the MoCap system. The contribution of each compartment (i.e., $\%RCp_{i}$, $\%RCa_{i}$, $\%AB_{i}$) is calculated as a percentage of the $V_{T}$ in each respiratory cycle:

(3) $$\%RCp_{i}=\frac{V_{RCp,i}}{V_{T}i};\%RCa_{i}=\frac{V_{RCa,i}}{V_{T}i};\%AB_{i}=\frac{V_{AB,i}}{V_{T}i}$$

The average percentage contributions (i.e., %RCp, %RCa, %AB) over the entire test were calculated using the following formula:

(4) $$\%RCp=\sum_{i=1}^{N}\frac{\%RCp_{i}}{i};\%RCa=\sum_{i=1}^{N}\frac{\%RCa_{i}}{i};\%AB=\sum_{i=1}^{N}\frac{\%AB_{i}}{i}$$

#### 2.4.6. Comparison Between Systems

To investigate the performance of the MoCap system in estimating $f_{R}$ and $V_{T}$ values, these values were compared against those recorded by the reference instrument. Correlation and Bland–Altman analyses were carried out by using all the data gathered from participants per $f_{R}$ and $V_{T}$, in quiet breathing and incremental tests. The Bland–Altman plot was used to compare the agreement between the two systems. The plot shows the differences between the measurements obtained by the two systems on the y-axis and the average of the measurements on the *x*-axis. This plot shows the agreement between the two systems in terms of accuracy (mean of the differences, MOD) and precision (limits of agreement, LOAs). These limits represent the range within which approximately 95% of the differences between the two methods are expected to fall. Moreover, we calculated the mean absolute error (MAE), mean percentage error (MAPE), and root mean square error (RMSE) as in the following formulas (Equations (5)–(7)):

(5) $$MAE=\frac{1}{n}\cdot \sum |x_{MoCap}-x_{reference}|$$

(6) $$MAPE=\frac{1}{n}\cdot \sum \frac{|x_{MoCap}-x_{reference}|}{x_{reference}}\cdot 100$$

(7) $$RMSE=\sqrt{\frac{1}{n}\cdot \sum {\left(x_{MoCap}-x_{reference}\right)}^{2}}$$

## 3. Results

### 3.1. Athletes During Incremental Test

**Figure 3:**
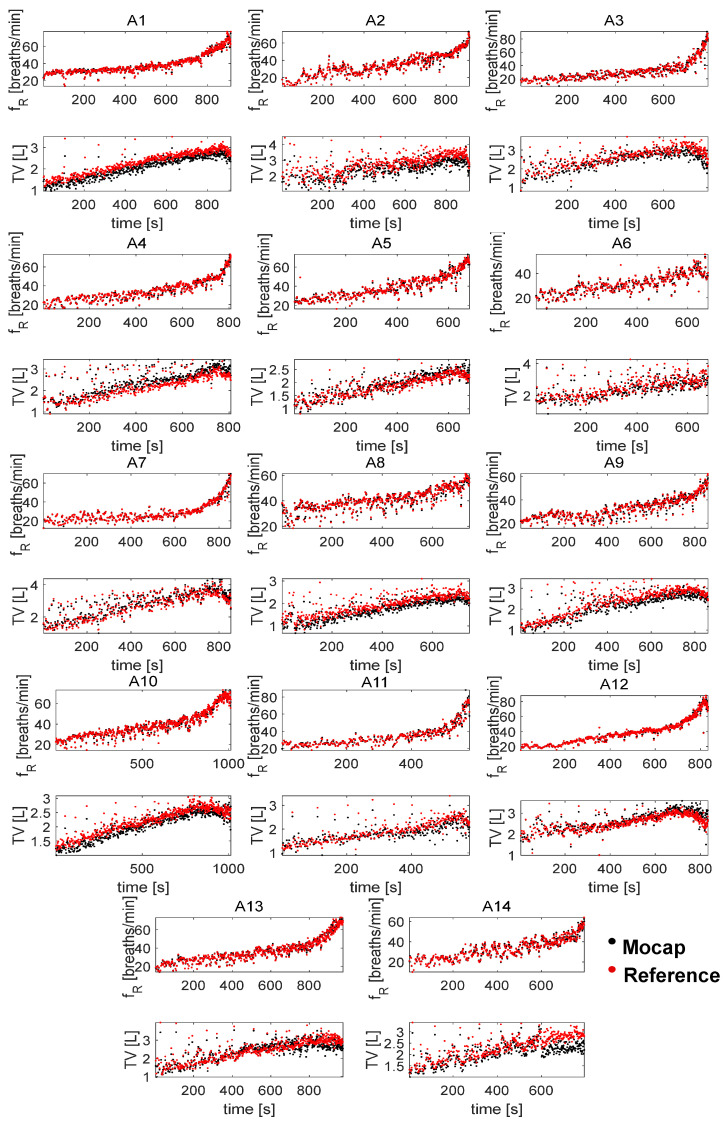
Time course of $f_{R}$ and $V_{T}$ breath-by-breath values for all 14 athletes analyzed during the incremental test. In black are the MoCap data, while in red are the reference values.

For the athlete population, the breath-by-breath time course of $f_{R}$ and $V_{T}$ recorded during the incremental test is shown in Figure 3 for each athlete. A nonlinear increase over time was generally observed for $f_{R}$ although with inter-individual differences in the $f_{R}$ time course. Both the MoCap and the reference systems showed a substantial decrease in $V_{T}$ in the final part of the test for most of the cyclists, as expected. The duration of the test and the number of breaths recorded differed for each athlete. The shortest test lasted 580 s (A11), and the longest test 1000 s (A10).

**Figure 4:**
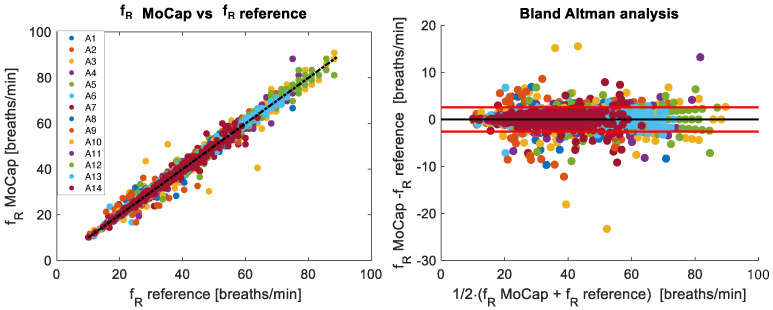
The (**left**) panel shows the correlation of breath-by-breath $f_{R}$ values between reference and MoCap systems for all 14 subjects analyzed during the incremental test. The Bland–Altman plot of the breath-by-breath $f_{R}$ values is depicted in the (**right**) panel. The black line represents the mean difference (MOD), while the red lines indicate ±1.96 standard deviations (LOAs).

**Figure 5:**
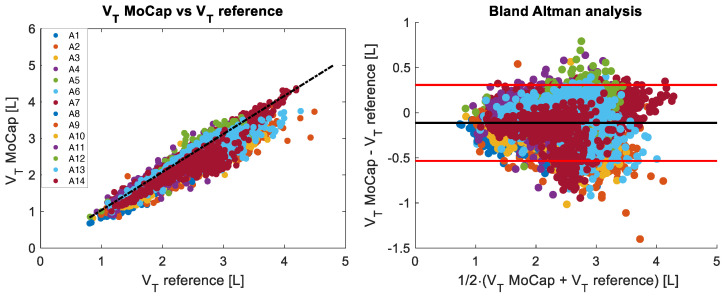
The (**left**) panel shows the correlation of breath-by-breath $V_{T}$ values between reference and MoCap systems for all 14 subjects analyzed during the incremental test. The Bland–Altman plot of the breath-by-breath $V_{T}$ values is depicted in the (**right**) panel. The black line represents the mean difference (MOD), while the red lines indicate ±1.96 standard deviations (LOAs).

Figure 4 and Figure 5 show the breath-by-breath comparison between the MoCap and reference systems of the recorded $f_{R}$ and $V_{T}$ values, respectively. The $f_{R}$ values are in the range of 10 breaths/min–88 breaths/min, while $V_{T}$ varies between 0.8 L and 4.5 L.

On 6416 breaths, the coefficient of determination ($r^{2}$) of the correlation plot was 0.99 (*p* < 0.001), with a MAE of 0.79 breaths/min, a MAPE of 2.1%, and an RMSE of 1.31 breaths/min. The Bland–Altman analysis identified a MOD of −0.01 breaths/min and LOAs of ±2.58 breaths/min.

For $V_{T}$ values, the $r^{2}$ of the correlation plot was 0.87 (*p* < 0.001), with a MAE of 0.19 L, a MAPE of 8%, and an RMSE of 0.21 L. The Bland–Altman analysis shows a bias (MOD ± LOA) of −0.11 ± 0.42 L.

**Figure 6:**
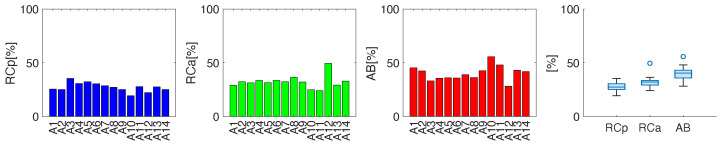
Average percentage contribution related to RCp, RCa and AB for the 14 athletes during the incremental test, and a boxplot summarizing the average percentage contribution of the overall population.

The data in Figure 6 show the average percentage contributions of the three compartments. In general, all subjects showed a higher contribution of the abdominal compartment except for subject A12, which showed a different respiratory dominance compared to the other athletes. The abdominal thoracic cage compartment was also prevalent over the pulmonary thoracic cage compartment, except for participants A3, A5, and A11. When assessing the compartmental contribution of all participants, a median percentage of 27.4%, 32.2%, and 40.4% was observed for RCp, RCa and AB, respectively.

**Figure 7:**
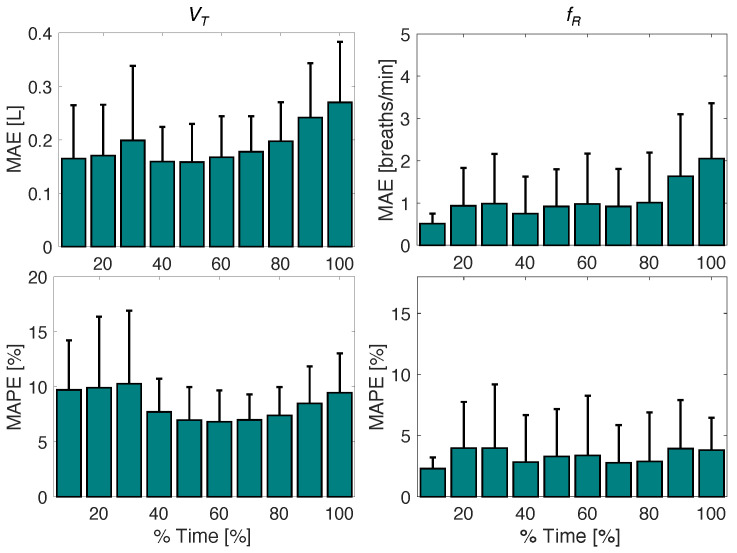
MAE and MAPE values with the relative uncertainty calculated for the estimated $f_{R}$ and $V_{T}$ per each of the 10 intervals of the incremental test considering all the athletes.

To better compare the values obtained by the athletes during the incremental test, Equations (5) and (6) were used to calculate MAE and MAPE values of 10 intervals for each 10% of the total test time. This analysis, shown in Figure 7, allows for a better appreciation of the errors obtained at low exercise intensities (first bars) and those at higher intensities (last bars). For $f_{R}$, MAE values showed an increase with exercise intensity at 90% and 100% of the test, but MAPE values remained relatively constant. For $V_{T}$, a slight increase in MAE was observed in the second half of the test, with minimum average MAE values of 0.16 L at 10% of the trial and maximum average values of 0.27 L at 100%. However, no substantial increase in MAPE was observed with the increase in exercise intensity.

### 3.2. Untrained Volunteers During Quiet Breathing

**Figure 8:**
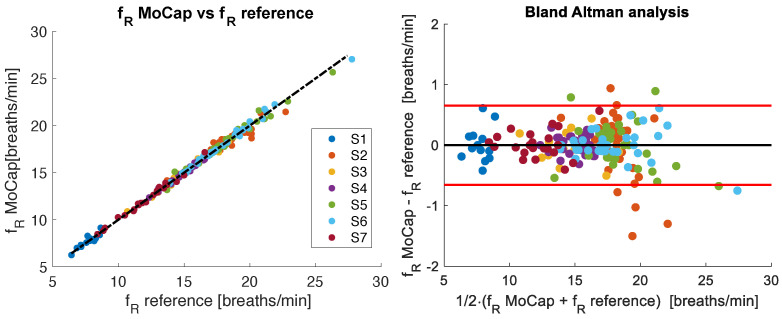
The (**left**) panel shows the correlation of breath-by-breath $f_{R}$ values between reference and MoCap systems for all seven untrained volunteers analyzed during quiet breathing. The Bland–Altman plot of the breath-by-breath $f_{R}$ values is depicted in the (**right**) panel. The black line represents the mean difference (MOD), while the red lines indicate ±1.96 standard deviations (LOAs).

Seven untrained volunteers were evaluated during a period of quiet breathing. For $f_{R}$, the $r^{2}$ of the correlation plot was 0.99 (*p* < 0.001), with a MAE of 0.23 breaths/min, a MAPE of 1.5%, and an RMSE of 0.09 breaths/min. The Bland–Altman plot presented in Figure 8 shows MOD of −0.01 breaths/min and LOAs of ±0.65 breaths/min.

**Figure 9:**
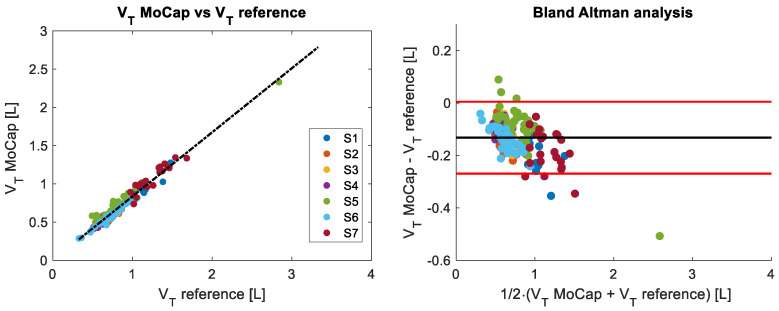
The (**left**) panel shows the correlation of breath-by-breath $V_{T}$ values between reference and MoCap systems for all seven untrained volunteers analyzed during quiet breathing. The Bland–Altman plot of the breath-by-breath $V_{T}$ values is depicted in the (**right**) panel. The black line represents the MOD, while the red lines the LOAs.

For $V_{T}$ the $r^{2}$ of the correlation plot was 0.96 (*p* < 0.001), with a MAE of 0.13 L, a MAPE of 16.5%, and an RMSE of 0.05 L. The Bland–Altman plot presented in Figure 9 shows a MOD of −0.13 L and LOAs of ±0.14 L. Therefore, the MoCap system slightly underestimates the $V_{T}$ values recorded with the reference system, but the dispersion is narrow enough that it does not compromise the analysis of $V_{T}$ during quiet breathing.

**Figure 10:**
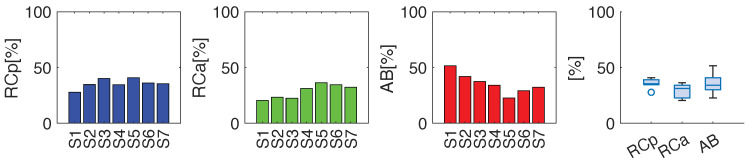
Average percentage contribution related to RCp, RCa and AB for the seven untrained volunteers during quiet breathing, and boxplot summarizing the average percentage contribution of the overall population.

Regarding breathing kinematics, the volunteers exhibited varying respiratory patterns. Overall, there was a general tendency to rely more on the thoracic compartment, as illustrated in Figure 10. However, some volunteers showed different dominant contributions—for example, in S3, the RCp compartment was the primary contributor, while in S1, the AB compartment played the leading role. Furthermore, the compartmental contribution of all participants results in a median percentage of 35.4%, 31.2% and 34.2% for RCp, RCa and AB, respectively.

## 4. Discussion and Conclusions

To the best of our knowledge, this is the first study validating the 32-marker protocol during exercise. The use of passive markers, affixed with hypoallergenic adhesive, do not interfere with the subject’s natural posture or breathing mechanics. This stands in contrast to flow meters or masks, which may alter respiratory patterns, especially during exercise. Moreover, unlike pneumotachographs or systems based on flow integration, the MoCap system is not affected by pressure drops or signal drift. This makes it ideal for long-duration monitoring, including real-time detection of dynamic hyperinflation and compartmental contributions. Furthermore, one of the major benefits of the MoCap-based system is the ability to analyze the contributions of RCp, RCa and AB separately—something not feasible with conventional spirometry or turbine-based systems. This capability enables a deeper understanding of breathing biomechanics, compensatory strategies, and muscle recruitment during physical effort.

Our experimental tests revealed good precision and accuracy of the MoCap system in the measurement of $f_{R}$, while an underestimation of $V_{T}$ was generally observed. Yet, the precision in the $V_{T}$ measurement was good, as outlined by the relatively narrow LOAs observed. These findings provide insight into the use of optoelectronic plethysmography during exercise.

An important methodological advantage of the present study is the breath-by-breath comparison made with the reference system, which substantially improves the more commonly used way to compare $f_{R}$ and $V_{T}$ values over windows of several seconds [24]. This is of note because the measurement error generally decreases as we move from a breath-by-breath comparison to a comparison made on progressively longer windows [24]. This issue must be considered when comparing the measurement error across different studies.

When measuring $f_{R}$, the 32-marker protocol showed good performance both at rest and during exercise. During quiet breathing, MAE was 0.23 breaths/min and MAPE was equal to 1.5%. During incremental exercise, we found a MAE value of 0.79 breaths/min and a MAPE value of 2.1%. Furthermore, no substantial increase in MAPE was observed during the incremental test, suggesting the measurement error is largely independent of exercise intensity. Previous validation studies of the MoCap system during exercise have mainly focused on the validation of $V_{T}$ rather than $f_{R}$, and the validation has rarely been performed on a breath-by-breath basis, hence limiting the comparison of the present findings with those of previous studies. However, the good performance of the MoCap system found in this study is further evident when considering that similar breath-by-breath error values were found when validating contact-based systems measuring variations in pressure or air temperature at the nose and/or mouth levels during cycling exercise [25,26].

When measuring $V_{T}$, the 32-marker protocol generally underestimated the values measured by the reference system, while relatively narrow LOAs were found. The Bland–Altman analysis showed MOD ± LOA values of −0.13 ± 0.14 L at rest and values of −0.11 ± 0.42 L during exercise, while average MAPE values were values were 16.5% and 8%, respectively. Importantly, MAPE did not show substantially increased values when comparing low-intensity and high-intensity exercise. The relatively good performance of the MoCap system during exercise is further outlined by the similar time course of $V_{T}$ found between the MoCap and reference systems for all the participants (see Figure 3), hence highlighting the suitability of the 32-marker protocol despite the underestimation observed. The systematic underestimation of $V_{T}$ observed with the 32-marker protocol can be attributed to several technical and methodological factors. One of the main causes is the limited spatial coverage offered by this reduced marker set compared to more comprehensive protocols, such as those using 86 [7] or 89 markers [15]. In particular, the 32-marker configuration does not include markers on cranial regions above the second rib, caudal abdominal areas below the umbilicus, or the lateral sides of the trunk, such as the axillary regions. These areas can significantly contribute to thoracic expansion during deep inspiration, and their exclusion inevitably results in a loss of volume-related information. As a consequence, the convex hull algorithm used to reconstruct thoracoabdominal volume may fail to fully capture the three-dimensional displacement of the chest wall, leading to consistent underestimation. Massaroni et al. [19] directly compared the 32-marker protocol with the 89-marker protocol at rest and found lower $V_{T}$ values with the 32-marker protocol, in line with the underestimation observed herein. Other studies have reported good precision and accuracy of the 86- or 89-marker protocols in measuring $V_{T}$ during exercise, but these assessments were typically limited to very short time windows (e.g., less than 60 s), which facilitated marker tracking under controlled conditions but did not reflect the demands of continuous monitoring. Providing average values over 10 breaths, Kipp et al. [27] found MOD ± LoA values of 0.01 ± 0.28 L during cycling exercise. By averaging values every minute, Layton et al. [16] showed MOD ± LoA values of 0.09 ± 0.10 L during cycling at maximal effort. However, we are not aware of any studies comparing breath-by-breath values of $V_{T}$ between a MoCap system and a reference system during several minutes of exercise. Additionally, as discussed elsewhere [28], the complexity of the 89-marker protocol limits its use in applied contexts and monitoring context. Although alternative techniques for measuring $V_{T}$ exist—such as wearable systems based on impedance pneumography [29] or piezoresistive sensors [30]—these typically require subject-specific calibration to achieve accurate estimations [29]. In contrast, the MoCap-based approach allows for subject-independent estimation, eliminating the need for individual calibration procedures [31]. Moreover, the results obtained in terms of MAPE and LOAs are comparable to, or even better than, those reported for some wearable devices, particularly during physical exercise, where dynamic conditions can adversely affect the performance of wearable sensors [32].

Monitoring the breathing pattern is gaining renewed attention during exercise in light of evidence suggesting that $f_{R}$ and $V_{T}$ are largely modulated by different inputs [33,34]. $f_{R}$ appears to be mainly modulated by fast inputs (e.g., central command and muscle afferent feedback), while $V_{T}$ appears to be fine-tuned based on $f_{R}$ levels and the magnitude of metabolic inputs [34]. In this perspective, MoCap offers several advantages in the attempt to understand how the ventilatory control system sets the breathing pattern observed during exercise. Indeed, important information provided by the MoCap system cannot be obtained with typically used flow meters. From breath-by-breath compartmental volumes, MoCap allows the user to assess the synchrony between the different compartments and to record end-inspiratory and end-expiratory lung volumes for each compartment, as the MoCap is not prone to signal drift, unlike the flow meter signal [27]. As such, MoCap has been used to assess the extent of dynamic hyperinflation during exercise or the effects of interventions (e.g., salbutamol, oxygen therapy, and real-time feedback) on the breathing pattern [28,35,36,37]. Some of these and other applications are favored with a 32-marker protocol that can more easily be implemented in applied settings than the traditional 89-marker protocol.

The use of a 32-marker protocol is especially required when athletes, patients, or practitioners are evaluated in applied contexts, where the cumbersome and time-consuming setup and data processing of more complex protocols cannot be afforded [28]. Furthermore, the 32-marker protocol allows for the reproduction of the classical posture of the athlete riding a bike or performing other activities. Hence, this is among the few studies that evaluated the performance of optoelectronic plethysmography with competitive cyclists exercising in their normal posture with their hands on the handlebars. Indeed, a common limitation of the MoCap system is that participants are usually forced to use unnatural body positions, typically involving extending their arms away from the torso to avoid obstructing the camera’s view of the markers [16]. As such, the 32-marker protocol neither alters the cyclist’s posture nor affects the breathing pattern because of the equipment used. Conversely, when measuring breathing variables with flow meters, the use of mouthpieces or masks may alter the breathing pattern [38,39,40,41].

In conclusion, the MoCap system is robust in estimating respiratory variables during exercise, making it a valuable tool for athletic and clinical settings. Its non-invasive nature and ability to capture compartmental volumes allow for continuous monitoring of respiratory dynamics. However, while the 32-marker protocol shows good performance in measuring $f_{R}$, some improvements in the measurement of $V_{T}$ are to be encouraged. A viable strategy to reduce the systematic error in the estimation of $V_{T}$ could be the application of a calibration algorithm based on least squares or robust regression at the beginning of each trial [42], minimizing the difference between MoCap and reference system volumes over an initial window (e.g., the first ten breaths). This correction factor could then be applied to the rest of the data, offering a computationally efficient way to improve accuracy while maintaining the practical advantages of the 32-marker setup for long-term monitoring. Further approaches could be oriented to a targeted expansion of the marker set. This may involve adding additional markers on key regions as the axillary lines, without significantly increasing the complexity of the setup or implementing more advanced geometric modeling approaches, such as spline-based surface fitting [43] or deformable mesh reconstruction [44] to better capture the real morphology of the thoracoabdominal wall during respiration. Overall, while further refinements are needed, MoCap remains a promising tool for comprehensive respiratory assessment in a variety of applications, with the potential to serve as the gold standard contactless technique for estimating temporal parameters and volumes during exercise.

## Data Availability

The data presented in this study are available upon request from the corresponding author. The data are not publicly available due to privacy reasons.

## Abbreviations

The following abbreviations are used in this manuscript:

AB	Abdomen
COSMED	Reference metabolic cart (Quark CPET)
$f_{R}$	Respiratory Frequency
MoCap	Motion capture system
RCp	Pulmonary rib cage
RCa	Abdominal rib cage
$V_{T}$	Tidal Volume
